# Neuromuscular Fatigue Is Not Different between Constant and Variable Frequency Stimulation

**DOI:** 10.1371/journal.pone.0084740

**Published:** 2014-01-02

**Authors:** Maria Papaiordanidou, Maxime Billot, Alain Varray, Alain Martin

**Affiliations:** 1 Aix-Marseille University, CNRS, ISM UMR 7287, Marseille, France; 2 Movement to Health Laboratory, Euromov, Montpellier 1 University, Montpellier, France; 3 GRAME, Faculté de Médecine, Département de Kinésiologie, Université Laval, Québec, Canada; 4 INSERM U1093 Cognition, Action et Plasticité Sensorimotrice, Université de Bourgogne, UFR STAPS, Dijon, France; The University of Queensland, Australia

## Abstract

This study compared fatigue development of the *triceps surae* induced by two electrical stimulation protocols composed of constant and variable frequency trains (CFTs, VFTs, 450 trains, 30 Hz, 167 ms ON, 500 ms OFF and 146 ms ON, 500 ms OFF respectively). For the VFTs protocol a doublet (100 Hz) was used at the beginning of each train. The intensity used evoked 30% of a maximal voluntary contraction (MVC) and was defined using CFTs. Neuromuscular tests were performed before and after each protocol. Changes in excitation-contraction coupling were assessed by analysing the M-wave [at rest (M_max_) and during MVC (M_sup_)] and associated peak twitch (Pt). H-reflex [at rest (H_max_) and during MVC (H_sup_)] and the motor evoked potential (MEP) during MVC were studied to assess spinal and corticospinal excitability of the *soleus* muscle. MVC decrease was similar between the protocols (−8%, *P*<0.05). M_max_, M_sup_ and Pt decreased after both protocols (*P*<0.01). H_max_/M_max_ was decreased (*P*<0.05), whereas H_sup_/M_sup_ and MEP/M_sup_ remained unchanged after both protocols. The results indicate that CFTs and VFTs gave rise to equivalent neuromuscular fatigue. This fatigue resulted from alterations taking place at the muscular level. The finding that cortical and spinal excitability remained unchanged during MVC indicates that spinal and/or supraspinal mechanisms were activated to compensate for the loss of spinal excitability at rest.

## Introduction

Electrical stimulation (ES) is a technique to evoke muscle contraction via the application of electrical current, and it has been used to strengthen muscles in both athletic training and rehabilitation programs for the last 40 years. Although ES is an effective stimulus to induce force increases [1], it presents an important limitation that hinders its wide acceptance, at least in the clinical context. Indeed, ES causes precocious and high neuromuscular fatigue development [2]. The rapid fatigue onset is due to the motor unit (MU) recruitment pattern, which differs from the pattern observed under voluntary contractions (synchronous, spatially fixed, random, for a review see [3]), giving rise to an exaggerated metabolic response [4].

In the rehabilitation context, ES is used to strengthen the muscles of patients with motor deficiencies (such as spinal cord injury or stroke) and to supplement for lost functions in these patients. Under these conditions, ES fatigue is superimposed on the great fatigability that these patients’ muscles already present [5]. Hence, research has focused on finding the stimulation protocol that would enhance muscle performance while minimizing the rapid fatigue onset. Constant and variable frequency trains (CFTs and VFTs) have been proposed and compared in the literature in terms of force production and fatigue development. The stimulation frequency for CFTs is often 30 Hz [6], whereas VFTs include a high frequency doublet (100 Hz) at the beginning of each train that takes advantage of the muscle catchlike property. The catchlike property [7] is the force augmentation that occurs when an initial, brief, high frequency burst (2–4 pulses) is included in the beginning of a subtetanic low frequency stimulation train [8]. It has been shown that VFTs evoke greater force than CFTs [9]. However, the results concerning the fatigability produced by these two stimulation patterns remain equivocal. Indeed, when CFTs and VFTs were matched for the number of pulses, muscle fatigue, as evidenced by the torque decrease at the end of the stimulation protocol, was reported to be greater following VFTs [10], comparable between the two stimulation protocols [11], or even smaller following VFTs [12]. Similarly, when protocols were matched for the induced force-time integral, no differences [13] or even decreased muscle fatigue following VFTs [12] were observed. Methodological choices (muscle group studied, supra- *vs* sub-maximal stimulation, nerve *vs* muscle stimulation) may be the origin of these discrepancies. Moreover, the fatigue in these previous studies was analyzed as a purely muscular phenomenon, without the investigation of neural mechanisms. Yet it is now widely acknowledged that ES, despite its application in the periphery of the neuromuscular system, solicits neural structures, and this has been observed after acute and chronic ES application [14]–[17]. Acute application, under both high (75 Hz) and low (30 Hz) frequency stimulation, resulted in the development of neuromuscular fatigue, quantified by the decrease in maximal voluntary contraction (MVC) torque, and attributed partly to an incapacity of the central nervous system to maximally drive the muscles. This incapacity was reported to be due to either a failure of spinal excitability [18] or a decrease in the neural drive reaching the motoneurons [15]; [17]. The authors attributed these findings to the solicitation of muscle afferents that convey signals to spinal and/or supraspinal centers, which in turn alter their output toward the muscle.

Given that modulation of stimulation parameters induces distinct neuromuscular adaptations [19], it can be expected that the fatigue origin would differ between CFTs and VFTs. The initial doublet used in VFTs may entail increased solicitation of muscular mechanisms, leading to a greater disturbance of the muscle’s chemical environment. This would induce enhanced activation of the small diameter muscle afferents that modulate central command and/or motoneuron response [20]; [21]. Under these conditions, greater neural adaptations following VFTs are thus probable; however, to our knowledge, this has never been examined.

This study compared the nature of the fatigue induced by two ES protocols (CFTs and VFTs), matched for the number of delivered pulses and applied on the *triceps surae* muscle of healthy subjects, with the objective of determining whether the stimulation pattern is a key factor of the induced neuromuscular adaptations. We hypothesized that the greater metabolic disturbance induced by VFTs would entail greater neuromuscular fatigue involving muscular as well as neural mechanisms.

## Materials and Methods

### Subjects and Ethics Statement

Ten healthy males (32±3.8 years, 176.7±2.2 cm) volunteered to participate in the present study. They were all physically active with no history of ankle injury and no recent lower limb surgery. After being informed about the study objectives and the potential risks associated with study participation, they all signed the consent form. The study was approved by the Scientific Committee of the Faculty of Sport Science of Burgundy University and was conducted in accordance with the principles of the declaration of Helsinki.

### Experimental Design

**Figure 1:**
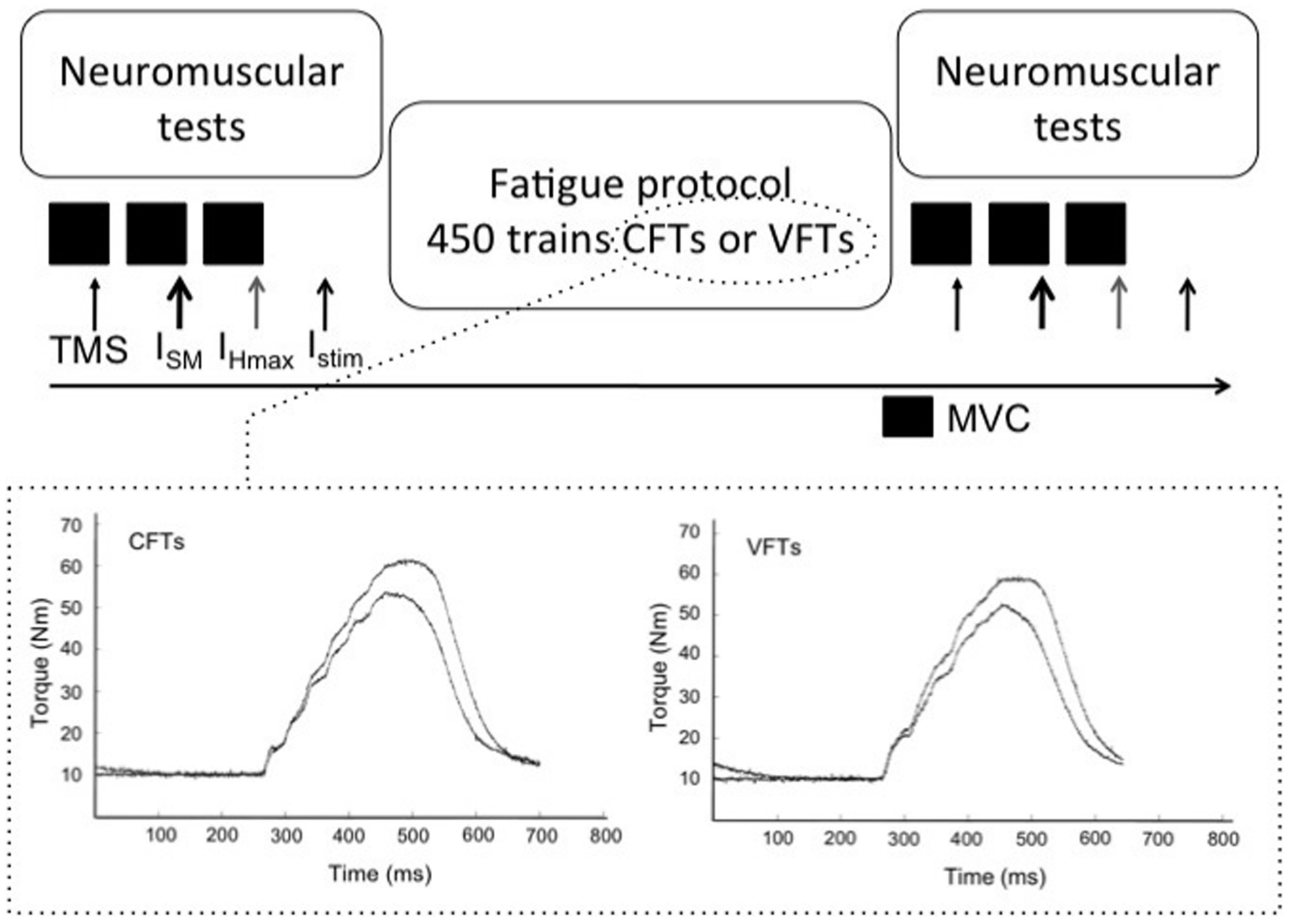
Schematic representation of the experimental protocol. Neuromuscular tests were performed before and after the fatiguing protocols, which were composed of 450 trains of 6 pulses (CFTs or VFTs according to the session). The difference between protocols was that stimulation trains for the VFTs protocol started with an initial doublet (100 Hz). The lower panel shows the torque response of a representative subject on the first and last train of stimulation, for the CFTs (left graph) and VFTs (right graph) protocols. During the neuromuscular tests, superimposed and at rest stimulations (indicated with arrows) were delivered in a random order. TMS: transcranial magnetic stimulation, I_SM_: stimulation at supramaximal intensity, I_Hmax_: stimulation at the intensity where H_max_ was observed, I_stim_: stimulation at the intensity used during the fatiguing protocol, MVC: maximal voluntary contraction.

Subjects visited the laboratory on one occasion and participated in two ES protocols, which were randomly assigned to the left and right lower limbs. One protocol was composed of constant frequency stimulation trains (CFTs), while the other included variable frequency trains (VFTs) (see fatigue protocols). Neuromuscular tests were conducted before and immediately after each of the stimulation protocols (Figure 1).

### Instrumentation and Procedures

#### Torque and EMG recordings

Torque during MVC and electrically evoked contractions was measured by an isokinetic dynamometer (Biodex 3, Shirley Corporation, NY, USA). Subjects were seated on the dynamometer chair with their foot strapped to the pedal, and straps were also applied to the chest and pelvis to secure positioning. The ankle joint was at 90° and the knee joint was at 110° (180° full extension) during the entire experiment. The dynamometer axis was aligned with the anatomical ankle plantar- and dorsi-flexion axis. To minimize head rotations that can affect cortico-vestibular influences on evoked potential recordings [22], subject’s head was secured in the same position by means of a rigid neck brace.

EMG activity of the *soleus* (SOL), *gastrocnemius medialis* (GM), *lateralis* (GL) and *tibialis anterior* (TA) muscles was recorded bipolarly by silver chloride square surface electrodes with a recording diameter of 9 mm (Contrôle Graphique Medical, Brie-Compte-Robert, France). In order to minimize impedance (<5 kΩ), the skin was shaved, abraded and cleaned with alcohol. Electrodes were placed over the middle of the muscle belly of the GM, GL and TA muscles, whereas the electrodes for SOL were placed along the mid-dorsal line of the leg, about 5 cm distally to the insertion point of the two *gastrocnemii* to the Achilles tendon. Electrode position was secured after verification of an appropriate M-wave acquisition (single response, highest amplitude for a given intensity). The ground electrode was placed on the contralateral patella and the inter-electrode distance for recording electrodes was 20 mm. Torque and EMG activity signals were synchronously recorded (acquisition frequency 5 kHz) and stored for analysis with commercially available software (Tida, Heka Elektronik, Lambrecht/Pfalz, Germany).

### Stimulation

Evoked potentials were elicited through electrical stimulation of the tibial nerve and magnetic stimulation over the motor cortex (M1), while electrical stimulation over the *triceps surae* muscle (myostimulation) was used to induce fatigue.

#### Tibial nerve stimulation

The tibial nerve was stimulated by means of a high-voltage, constant-current stimulator (DS7AH, Digitimer, Hertfordshire, UK), delivering monophasic, rectangular, 1-ms pulses. The anode (10×5 cm) was placed beneath the patella and, after verification of an appropriate H-reflex acquisition in the SOL muscle, the cathode (a silver chloride surface electrode, 9-mm diameter) was fixed in the popliteal fossa. Stimulation intensity was increased by 4-mA increments from H-reflex threshold until no further increase in the M-wave amplitude of the SOL muscle was observed. This intensity was further increased by 10% (I_SM_) and was maintained for the entire testing procedure. Parallel to the M-wave recruitment curve, the H-reflex was also recorded. Subsequently, the intensity where the maximum H-reflex was observed was carefully sought (I_Hmax_) using 1-mA increments. For these recruitment curves, four stimulations were delivered at each intensity every 10 s.

#### Transcranial magnetic stimulation (TMS)

An eight-shaped coil (70 mm diameter, power output 2.6 T) positioned over the motor cortex was used to elicit motor evoked potentials (MEPs) in the SOL muscle in response to TMS (Magstim 200 stimulator, Dyfed, UK). To find the greatest amplitude for the SOL evoked response with the lowest stimulation intensity, the M1 area of the *triceps surae* muscle was stimulated by starting from 1 cm posterior and 1 cm lateral to the vertex of the subject's head. The optimal stimulation site was marked over the scalp to ensure reproducibility of the stimulation conditions for each subject throughout the experimental sessions. The coil was then secured in place throughout the experiment and orientated to deliver anterior-posterior directed stimulation to the brain. A MEP recruitment curve was performed. Because MEPs can rarely be recorded in lower limb muscles in resting conditions [23], subjects were asked to perform a submaximal voluntary contraction of the plantar flexors (30% of maximal isometric torque) with the aid of visual feedback. During these submaximal isometric contractions, stimulation intensity was increased by 5% increments of the maximal stimulator output, from motor threshold until the maximal MEP amplitude was obtained (mean 92.8±8.7% and 91.4±9.4% of the maximal stimulator output for CFTs and VFTs, respectively). Three stimulations were delivered at each intensity during each ∼20-s submaximal contraction.

#### Fatigue protocols

Two rectangular electrodes (10×5 cm) were used for the myostimulation fatiguing protocols of the *triceps surae*. One was placed 5 cm beneath the popliteal fossa and the other on the insertion point of the two heads of the *gastrocnemii* to the Achilles tendon. Stimulation trains were delivered by a Digitimer stimulator (DS7AH, Digitimer, Hertfordshire, UK). According to the experimental session, the *triceps surae* muscle was stimulated with CFTs or VFTs. Both protocols were composed of 450 trains. Each train included six pulses (pulse width of 500 µs). Pulses for the CFTs were delivered at 30 Hz, 167 ms ON, 500 ms OFF. The stimulation trains for the VFTs session consisted of six pulses, starting with a doublet at 100 Hz, followed by four pulses at 30 Hz (146 ms train duration). Trains were delivered at an intensity evoking 30% of MVC (mean values of the MVCs performed before the ES session). This intensity (I_stim_) was defined using CFTs and was kept constant throughout the two sessions.

### Neuromuscular Tests

Neuromuscular tests were performed before and immediately after each of the ES protocols. They were composed of voluntary and evoked contractions. For the MVCs, subjects were asked to maintain a maximal voluntary effort for 4 s. A twitch at I_SM_ was delivered when the plateau was reached (superimposed twitch), as well as 5 s after the end of the MVC (control twitch). Twitches evoked at I_SM_ and I_Hmax_ were delivered at rest and during MVC, while TMS was delivered only during MVC. A twitch at the intensity used in the fatiguing protocols (twitch at I_stim_) was delivered via myostimulation at rest. The order of the neuromuscular tests was randomly assigned during the pre and post conditions.

### Data Analysis

#### Torque analysis

Torque evoked during MVC, trains of stimulation, and twitches at I_SM_ and I_stim_ were recorded and analyzed offline. During MVC, the 500-ms duration frame prior to the stimulation and the amplitudes of the superimposed and control twitches were studied. The level of voluntary activation (VA) was calculated as follows [24]:



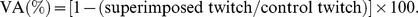



Peak torque and the force-time integral (FTI) during the stimulation trains were calculated as the average of 50 trains. This corresponded to a total of 9 sets of 50 trains for each 450-train protocol.

The mechanical response associated with twitches at I_SM_ was analyzed for the following contractile parameters: peak twitch (Pt), defined as the maximum value of torque production to a twitch, and contraction time, defined as the time between the beginning of the contraction until Pt. The amplitude of the twitch elicited at I_stim_ was analyzed.

#### EMG analysis

The evoked potentials obtained at rest (*i.e.* M_max_ and H_max_) and during MVC (*i.e.* M_sup_, H_sup_ and MEP) were analyzed for the peak-to-peak amplitude and area, defined as the signal integral between two cursors set at the start and the end of the potentials. Because both parameters showed similar changes, only peak-to-peak amplitude results are reported. The EMG activity associated with the twitches elicited at I_stim_ was also analyzed. H_max_ amplitude was normalized with respect to M_max_ (i.e., H_max_/M_max_ ratio) to avoid any impact of changes in membrane excitability. In the same way, H_sup_ and MEPs were normalized with respect to M_sup_ (i.e., H_sup_/M_sup_ and MEP/M_sup_ ratios). Despite the fact that the stimulation site for the twitch elicited at I_stim_ and the M_max_ was different (myostimulation *vs.* nerve stimulation), we also normalized electrophysiological responses of the twitch at I_stim_ with respect to M_max_. The silent period associated to MEP recording was measured during MVC as the interval from the stimulus artifact to the return of continuous EMG activity. The end of the silent period was determined when the corresponding rectified EMG activity reached the mean value extended by two standard deviations of the rectified EMG signal recorded for 1 s when subjects were at rest before the contraction. The EMG activity of the SOL, GM and GL muscles was quantified by the root mean square (RMS) value of the filtered signal (10–500 Hz) at the 500-ms interval used for MVC torque analysis. The RMS value was subsequently normalized to M_sup_ (RMS/M_sup_).

### Statistical Analysis

The normality of the data was verified using the Kolmogorov-Smirnov test. All variables measured before (pre) and after (post) each experimental session were tested using a two-way (protocol and time) repeated measures ANOVA. In the case of a significant main simple (time and protocol) or interaction effect (protocol×time), the LSD Fisher post-hoc test was used. The correlation between selected variables was tested with the Pearson coefficient. Data are reported in the text as means and standard deviation (SD) and the level of significance was set at *P*<0.05. In the case of no significant interaction effect, pooled data are presented. All tests were performed using Statistica software (StatSoft, Inc., version 7.1, Tulsa, OK).

## Results

### Fatiguing Protocols

**Figure 2:**
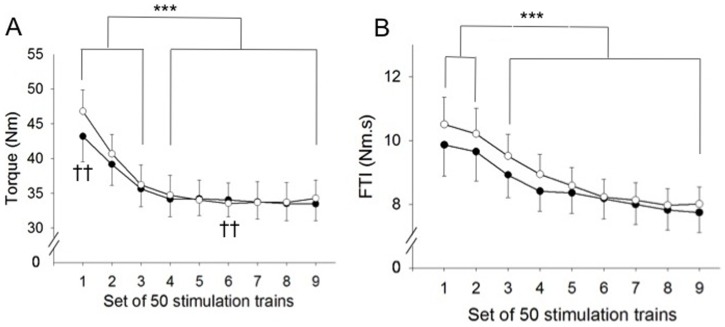
Characteristics of the fatiguing protocols. White circles correspond to VFTs (variable frequency trains) and black circles represent CFTs (constant frequency trains). A. Torque time-course during the nine 50-train bouts. Torque significantly decreased for both protocols from the second 50-train set. Torque evoked during the first 50-train bout was significantly higher for VFTs. B. FTI (force-time integral) evolution during the protocols. FTI significantly decreased throughout both fatiguing protocols. ††*P*<0.01 significant difference between protocols, and ***P<0.001 significantly different from first bout values. Values are presented as means ± SEM.

Statistical analysis revealed a significant interaction effect (protocol×time) for the developed torque (*P*<0.05). Although the same stimulation intensity was used in the two protocols, torque evoked during the first 50-train bout was significantly higher for VFTs than CFTs (46.78±9.17 Nm and 43.17±11.57 Nm, respectively, *P*<0.01). After the first bout, torque evoked by the two stimulation protocols progressively decreased until the fourth 50-train bout. Thereafter it remained stable until the end of the protocols, reaching a value of 34.25±7.81 Nm and 33.45±7.55 Nm after the 450 trains for VFTs and CFTs, respectively (Figure 2A). Relative torque decrease was significantly higher for the VFTs (25±11.82% *vs* 21±8.35%, *P*<0.05).

There was a time effect for FTI developed during the stimulation trains and this value significantly decreased after both protocols (*P*<0.001). From the initial value of 10.18±2.81 Nm.s, FTI decreased to 7.87±1.78 Nm.s at the end of the protocols (Figure 2B).

### Neuromuscular Tests

#### Maximal voluntary contractions (MVC)

Initial MVC values were not significantly different between protocols. There was a significant time effect on the MVC torque (*P*<0.05) after both protocols. From the initial value of 138.81±29.47 Nm, maximal voluntary torque decreased to 127.69±28.71 Nm at the end of the protocols, representing an ∼ 8% decrease. This decrease was not accompanied by changes in the level of voluntary activation, which remained stable after the two protocols (95.62±2.74% and 95.36±2.2% before and at the end of the protocols, respectively, *P* = 0.31). Similarly, SOL RMS/M_sup_ remained unaffected by the stimulation protocols (0.086±0.026 and 0.096±0.057 from pre to post values, respectively, *P* = 0.55). Similar results were obtained for GM and GL muscles (0.034±0.020 to 0.031±0.012 and 0.049±0.030 to 0.052±0.026 from pre to post values for GM and GL, respectively).

#### Electrically evoked contractions

Pt and twitch at I_stim_.

For the following parameters there was no significant difference between protocols. Pt significantly decreased after both protocols (from 25.44±5.40 Nm to 23.61±5.06 Nm for pre and post values, respectively, *P*<0.05), whereas contraction time was not affected by the fatiguing exercise (*P = *0.24).

**Figure 3:**
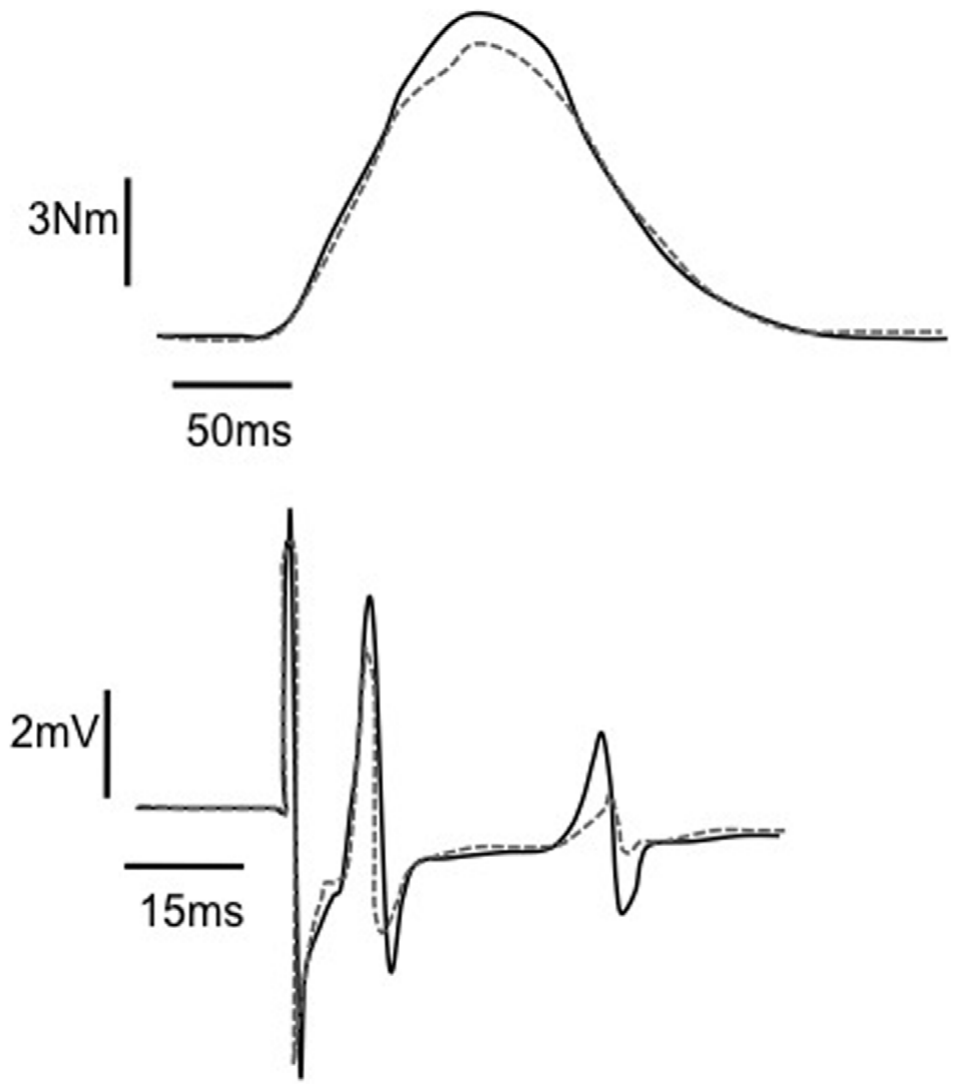
Typical traces of the twitch elicited at the CFTs intensity from a representative subject. Solid black lines correspond to recordings obtained before the protocol and dashed grey lines show the post-exercise recording. The upper panel depicts the mechanical response, and the lower one presents the electrophysiological responses. Note that for this subject, stimulation intensity evoked M-wave and H-reflex responses. Both mechanical and electrophysiological responses decreased at the post-stimulation stage.

Figure 3 (upper panel) represents the twitch at I_stim_ of a characteristic subject before and after the CFTs protocol. A significant time effect was observed for the twitch elicited at I_stim_ (*P*<0.01) for both protocols. From the initial value of 11.09±2.89 Nm, twitch amplitude decreased to 9.09±2.13 Nm at the end of the protocols, representing a 20% decrease. This decrease was positively correlated with the torque decrease observed during the stimulation trains (r = 0.67, *P*<0.05).

#### Electrophysiological responses

**Figure 4:**
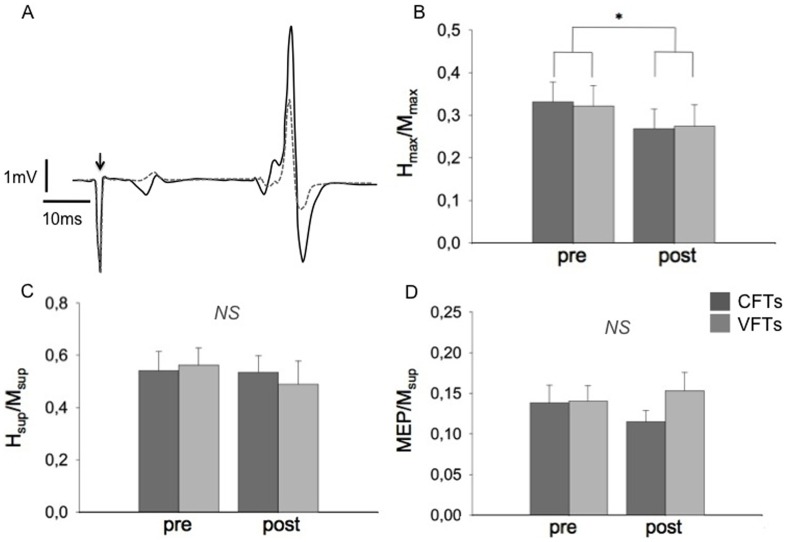
Electrophysiological responses at pre- and post-fatigue conditions. A. Typical recording of the H_max_ response of a representative subject. H_max_ was elicited at rest and was accompanied by a submaximal M-wave. The arrow indicates time of stimulation. The solid black line corresponds to pre-fatigue and the dashed grey line represents post-fatigue measurement. B, C and D show ratios (mean ± SEM) of H_max_/M_max_, H_sup_/M_sup_ and MEP/M_sup_ respectively, obtained before and after the stimulation protocols. Dark grey bars represent the CFTs protocol while grey bars the VFTs protocol. * *P*<0.05 significant pre-post effect, NS not statistically significant.

M-wave amplitude significantly decreased at rest (*P*<0.01) and superimposed to MVC (*P*<0.05) after both protocols. The H_max_/M_max_ ratio at rest (see Figure 4A for a typical recording of H_max_) significantly decreased after the two stimulation protocols (−21%, *P*<0.05), whereas it remained unchanged when elicited during MVC (*P* = 0.38). The M-wave amplitude accompanying both H_max_ and H_sup_ normalized to M_max_ and M_sup_ (i.e., M_atHmax_/M_max_ and M_atHsup_/M_sup_ respectively) remained stable after both protocols (*P* = 0.56 and *P* = 0.49), ensuring that reflex results were not affected by the stimulation conditions. MEP/M_sup_ and the silent period were not significantly affected by the stimulation protocols (*P* = 0.97 for MEP/M_sup_ and *P* = 0.51 for the silent period). Analysis of the electrophysiological responses obtained during the twitch at I_stim_ revealed that, for all subjects, M-waves and H-reflexes were present (M_Istim_ and H_Istim_) but their amplitude was not significantly affected by the stimulation protocols (*P* = 0.3 and *P* = 0.08 for M waves and H reflexes respectively). Recordings of a representative subject are presented in Figure 3B. In Table 1, the electrophysiological responses are presented, while Figure 4 shows the evolution of the electrophysiological ratios.

**Table 1:** Electrophysiological responses before and after the two stimulation protocols.

	pre	post
M_max_ *(mV)*	10.59±2.45	9.13±2.49**
M_sup_ *(mV)*	12.50±3.59	10.44±3.96*
H_max_ *(mV)*	3.62±1.96	2.58±1.86*
H_sup_ *(mV)*	7.07±3.95	6.00±3.96*
MEP *(mV)*	1.60±0.70	1.23±0.49*
silent *(ms)*	128±15	123±19
M_atHmax_/M_max_	0.11±0.12	0.11±0.14
M_atHsup_/M_sup_	0.15±0.17	0.16±0.2
M_Istim_/M_max_	0.36±0.18	0.29±0.15
H_Istim_/M_max_	0.063±0.064	0.038±0.027

M_max_: maximal M wave at rest, M_sup_: maximal M wave elicited during MVC, H_max_: maximal H-reflex at rest, H_sup_: maximal H reflex elicited during MVC, MEP: motor evoked potential elicited during MVC, silent: silent period accompanying MEP during MVC. M_Istim_ and H_Istim_: M wave and H-reflex obtained during twitch evoked at the stimulation intensity.

*P*<0.05,

*P*<0.01 significantly different from pre- values. Values are presented as mean ± SD (pooled data).

## Discussion

The aim of the present study was to compare neuromuscular adaptations after an acute application of two ES protocols, one composed of trains delivered at constant frequency (CFTs) and one including a doublet at the beginning of each stimulation train (VFTs). The main results showed no differences in the development of neuromuscular fatigue between the two protocols, as evidenced by similar MVC decreases and identical involvement of neuromuscular mechanisms. Indeed, the MVC decrease could be attributed to changes taking place distally to the neuromuscular junction (failure of the excitation-contraction coupling), while at the neural level, decreased spinal excitability was observed only at rest after both protocols.

In order to compare CFTs and VFTs, we chose to match the two protocols for the number of delivered pulses. The fatiguing protocols consisted of 450 trains of six pulses either at a constant frequency or delivered with an initial doublet followed by four pulses at a constant frequency. Moreover, the two protocols were evoked at the same intensity (30% MVC), thus ensuring recruitment of a similar motor unit pool. Hence, the only initial difference between protocols was the recruitment pattern of the solicited MUs. Under these conditions, VFTs evoked a greater torque in the initial phase of the protocol (8% higher than CFTs) and a greater relative torque decrease during the stimulation trains (25% *vs* 21% for VFTs and CFTs, respectively). Therefore, in the clinical context, when the aim of ES is to achieve high torque levels in order to accomplish the desired functional movement, VFTs appear more advantageous than CFTs protocols. This finding, in addition with observations that VFTs can enhance force output when muscles are in the fatigue state [10] and hence prolong the task, show the important functional implications of such stimulation pattern.

The enhanced torque development during VFTs was attributed to the initial doublet that takes advantage of the muscle catchlike property, which involves increased sarcoplasmic Ca^2+^ concentration and enhanced stiffness of the series elastic component of the muscle [8]. However, this enhancement was only observed at the start of the fatiguing protocol and did not significantly affect total FTI, which was not statistically different between the two protocols. The number of delivered stimulation trains being considerable (450 trains), the mechanisms responsible for the initial torque increase during the VFTs protocol (i.e. increased Ca^2+^ concentration and/or increased muscle stiffness) must have been compromised and surpassed by the development of muscle fatigue. Our results do not allow to specifically determine the depressed mechanism, but show the transient character of the catchlike phenomenon that did not differentially impact the subjects’ capacity to generate maximal voluntary force. Our initial hypothesis was that VFTs would induce greater fatigue development due to an increased metabolic requirement of the muscle [11]. It appears that a single doublet at the beginning of each train did not provide enough stress to induce metabolic changes distinct from those of CFTs to influence neuromuscular fatigue development. This was further reinforced by the finding that qualitatively the fatigue did not differ between protocols. In fact, the development of neuromuscular fatigue after both protocols could be attributed to the same muscular and neural mechanisms. Therefore, for the present study, the stimulation pattern used to recruit MUs did not seem to be the main parameter determining the amount or the nature of fatigue.

The significant decrease in the subjects’ maximal torque-generating capacity after the fatiguing ES protocols was accompanied by alterations taking place at the muscular level. A significant decrease in M_max_ amplitude was observed, giving evidence of changes in muscle excitability most probably due to impaired Na^+^/K^+^ pump function [25]. This dysfunction is known to lead to increased K^+^ efflux to the extracellular space concomitantly to increased intracellular Na^+^ income, both of which have been associated with a failure of excitation and a reduction in force [26]. In tandem to these changes, we observed a decrease in the amplitude of the potentiated twitch (Pt), indicating altered muscle contractile properties. Muscle contractile properties, which are related to Ca^2+^ kinetics beyond the muscle cell membrane [27], are sensitive to muscle’s metabolic environment. Increased myoplasmic inorganic phosphate concentration ([Pi]) has been presented as a potential mechanism for alterations in muscle contractile capacity. [Pi] has been shown to act directly on the cross-bridge function [28] or indirectly by reducing Ca^2+^ availability for the cross-bridge binding [29]; [30]. In addition to [Pi]-induced disturbances, other metabolic factors, such as excessive reactive oxygen species production, ATP or Mg^2+^, have also been proposed to play a role in reducing force production in repetitively activated muscles [26]. These mechanisms do not exclude that the twitch response decrease observed in the present study could also be linked to modifications of the mechanical properties of the muscle-tendon unit [8].

Parallel to the alterations in muscle excitability and twitch response, we observed for the first time that the amplitude of the muscle twitch elicited at the intensity used during the ES protocols was decreased under post-fatigue conditions. This twitch was an index of the mechanical behavior of the MUs solicited during the stimulation protocols. This finding was further confirmed by the significant correlation between torque decrease during the stimulation trains and the decrease in the amplitude of the muscle twitch. However, the torque decreases during the stimulation protocols (∼−20%) and at the twitch elicited at I_stim_ (∼−17%) were of greater amplitude compared with the one observed for Pt (∼−8%). This difference suggests that the torque decrease during the stimulation trains should not be attributed only to the fatigue of the solicited MUs but also to the loss of recruited MUs. Indeed, repetitive electrical activation of a muscle can lead to an increase in the excitability threshold of active motor axons. Motor axons having an excitability threshold close to the stimulation intensity can be lost during elicited contractions, thus contributing to the torque decrease [31]; [32]. Based on the results of the electrophysiological responses associated with the twitch at I_stim_ observed under post-fatigue conditions (Figure 3, Table 1), this latter phenomenon cannot be excluded. Moreover, these electrophysiological responses provide insight into the way by which MUs are activated during ES. It is now acknowledged that during ES muscle fibers can be recruited directly, through motor axon depolarization, as well as indirectly, through activation of spinal motoneurons by the afferent volley evoked in the mixed intramuscular axonal branches [33]; [34]. Direct recruitment can be identified by the M-wave, while H-reflex represents the MUs indirectly activated. Electrophysiological analysis of the muscle twitch revealed both direct and indirect activation of the MUs during the present ES protocols (Figure 3, Table 1). The repetitive activation of spinal loops under these conditions can partly contribute to the result concerning spinal excitability in our study.

Indeed, after both protocols, spinal excitability at rest decreased, as evidenced by the significant reduction in the H_max_/M_max_ ratio. Spinal excitability has rarely been reported to undergo changes after a fatiguing ES protocol. Butler and Thomas [35], using F-wave analysis, reported a reduction in motoneuron excitability following repetitive electrical activation of the upper limb, both in control and spinal cord injured subjects. Moreover, the *abductor pollicis brevis* H/M ratio was shown to decrease after intermittent ES, a reduction possibly linked to increased presynaptic inhibition, activated by metabolic changes in the activated muscle [18]. Presynaptic inhibitory mechanisms include homosynaptic post-activation depression of the Ia terminals (HPAD) [36] and primary afferent depolarizing interneurons (PAD) [37] that are both activated by Ia afferent repetitive discharge. Whatever the responsible mechanism (motoneuron excitability, HPAD, PAD), the results of these two studies, in tandem with the current finding that the MUs were partly activated indirectly via spinal loops under the present ES protocols, suggest that the observed decreased spinal excitability can be attributed to repetitive solicitation of the spinal loop leading to modifications in Ia afferents – motoneuron transmission [36]; [37]. Hence, the way by which MUs are activated during ES (direct or indirect activation) appears to be a determining parameter of the fatigue-induced neural adaptations.

Despite the spinal excitability modifications at rest, the VA and RMS/M_sup_ obtained during MVC were not affected by the fatiguing protocols, reinforcing the muscular origin of the induced fatigue. Moreover, H_sup_/M_sup_, MEP/M_sup_ and the silent period were not significantly changed in post-fatigue conditions, suggesting that the neural drive reaching the *triceps surae* was unaffected by the fatiguing exercise. However, the preserved spinal excitability during voluntary contraction in combination with the loss of spinal excitability at rest suggests that at post-fatigue conditions neural mechanisms were activated in order to compensate for this loss. Possible neural mechanisms include increased neural drive to the motoneuron pool and modulation of pre- or postsynaptic inhibition. These can be addressed to the spinal level via cortical and/or peripheral inputs. Despite the fact that our experimental design does not allow to distinguish between the responsible mechanisms, the preserved spinal excitability during MVC under post-exercise conditions suggests a modification in the relative contributions of spinal and supraspinal mechanisms in order to compensate for the decreased spinal excitability at rest.

In conclusion, the results of the present study show that fatigue development under ES is not dependent on the stimulation pattern of the solicited MUs, since CFTs and VFTs matched for the number of delivered pulses induced the same decreases in the MVC torque. Moreover, these decreases were attributed to muscular fatigue mechanisms (failure of excitation-contraction coupling). Despite the finding that neural mechanisms did not significantly contribute to the maximal torque decreases, specific neural adaptations were observed after both protocols. In fact, the indirect MU activation during ES may have caused spinal excitability depression at rest, which was then compensated by neural mechanisms activated during voluntary contraction. It is suggested that acute ES neuromuscular adaptations are more dependent on the way by which MUs are recruited during ES than on the stimulation pattern of the recruited MUs used in the present study.
